# A New Chief Editor for *Neural Plasticity* Growth

**DOI:** 10.1155/2020/1351395

**Published:** 2020-08-01

**Authors:** Michel Baudry

**Affiliations:** Western University of Health Sciences, Pomona, CA, USA

## Abstract

*Neural Plasticity* is pleased to announce the appointment of Dr. Michel Baudry as its new Chief Editor. Dr. Baudry is currently University Professor at Western University of Health Sciences in Pomona, CA. In this Editorial, Dr. Baudry describes some of the journal's journey and current status, and shares his vision and aspirations for its future.

*Neural Plasticity* has turned 21! Since its renaming in 1998, the journal has matured and has become a significant player in the field of neurosciences. While the term plasticity is generally attributed to William James in his *Principles of Psychology* [1], the term neuroplasticity appears to have been used first by Jerzy Konorsky in the 1940s [2]. Over the years, the plasticity of the nervous system has been invoked to account for a large variety of phenomena related to the ability of the brain to modify synapses, neurons, and neural circuits to adapt to changes in environment, stimuli, and life-related events. *Neural Plasticity* publishes articles related to all aspects of neural plasticity, with special emphasis on its functional significance as reflected in behavior and in psychopathology. The journal publishes both research and review articles from the entire range of relevant disciplines, including basic neuroscience, behavioral neuroscience, cognitive neuroscience, biological psychology, and biological psychiatry. Thus, the journal covers all aspects of plasticity, including molecular plasticity, synaptic plasticity, and plasticity at the levels of neurons and neural circuitries taking place under physiological or pathological conditions.

We are entering an exciting period, as the technological progress is allowing us to perform experiments that could not have been possible 20 years ago. To name a few, it is now possible to use minimicroscopes to record the activity of large ensembles of neurons for months and to analyze how behavior can affect neuronal activity. Optogenetics provides a tool to manipulate the activity of selected groups of neurons to help understand the functions of these neurons. Minibrains represent three-dimensional organoids generated from human cells reprogrammed to become neurons, which can mimic the brain status from an infinite variety of brain disorders. The brain computer interface popularized by the movie “The Matrix” [3] is becoming a reality, and several biotech companies are already attempting to develop applications for such interfaces.

However, as I reflect on the state of the field 20 years ago, I am also struck by the fact that, for most of the fundamental questions we were asking then, we still do not have answers. We are still struggling with many issues, including the links between synaptic plasticity and learning and memory, the roles of the multiple mechanisms of plasticity in various forms of behavioral plasticity, and how we can use our knowledge to restore defects in plasticity present in multiple pathological conditions. We need to remind ourselves that conceptual advances are as important as technological advances to move the field forward. We also need to remind ourselves that scientists have the responsibility to educate the public. As Carl Sagan said, “We live in a society exquisitely dependent on science and technology, in which hardly anyone knows anything about science and technology” [4]. In this regard, *Neural Plasticity* is proud to be part of the family of Open Access journals, which means that its content is accessible by anyone. Therefore, all of us, scientists, writers, and publishers, need to make a conscious effort to write in a way that is accessible to the majority of people and not to a restricted audience. We also have to make the significance of the research being published explicit and clearly understandable by the public. This will be a guiding principle in my new function as a Chief Editor of *Neural Plasticity*. I am looking forward to leading the journal in its next phase of growth and to work with the publisher, the editors, and the authors to make *Neural Plasticity* a leading journal in the field.

*Dr. Michel Baudry is currently University Professor at Western University of Health Sciences in Pomona, CA. His previous position was Professor of Biological Sciences, Neurology, and Biomedical Engineering at USC, Los Angeles, CA. After graduating from the prestigious Ecole Polytechnique in Paris (France) in 1971, Dr. Baudry obtained a Ph.D. in Biochemistry at the University of Paris VII in 1977. In collaboration with Professor Gary Lynch, he published in Science a new mechanism for learning and memory, which remains a landmark in the field. Since then, he has continued to work on understanding the molecular/cellular mechanisms of learning and memory and neurodegeneration.*

*Michel Baudry*
